# 5-Aminolevulinic Acid Triggered by Ultrasound Halts Tumor Proliferation in a Syngeneic Model of Breast Cancer

**DOI:** 10.3390/ph14100972

**Published:** 2021-09-25

**Authors:** Federica Foglietta, Giulia Gola, Elena Biasibetti, Maria Teresa Capucchio, Iside Bruni, Andrea Francovich, Gianni Durando, Loredana Serpe, Roberto Canaparo

**Affiliations:** 1 Department of Drug Science and Technology, University of Torino, 10125 Torino, Italy; federica.foglietta@unito.it (F.F.); giulia.gola.3@gmail.com (G.G.); roberto.canaparo@unito.it (R.C.); 2 Histopathology Department CIBA, Istituto Zooprofilattico Sperimentale di Piemonte, Liguria e Valle d’Aosta, 10154 Torino, Italy; elena.biasibetti@hotmail.it; 3 Department of Veterinary Sciences, University of Torino, 10095 Grugliasco, Italy; mariateresa.capucchio@unito.it (M.T.C.); iside.bruni@edu.unito.it (I.B.); 4 Institut de Physiologie, Université de Fribourg, 1770 Fribourg, Switzerland; andrea.francovich@unifr.ch; 5 National Institute of Metrological Research (INRIM), 10135 Torino, Italy; g.durando@inrim.it

**Keywords:** 5-aminolevulinic acid, ultrasound, sonodynamic therapy, breast cancer

## Abstract

Sonodynamic therapy is a bimodal therapeutic approach in which a chemical compound and ultrasound (US) synergistically act to elicit oxidative damage, triggering cancer cell death. Despite encouraging results, mainly for anticancer treatment, sonodynamics is still far from having a clinical application. Therefore, to close the gap between the bench and bedside, more in vivo studies are needed. In this investigation, the combined effect of 5-aminolevulinic acid (Ala), a natural porphyrin precursor, plus exposure to US, was investigated in vivo on a syngeneic breast cancer model. Real-time RT-PCR, Western blotting, and immunohistochemistry assays were performed to evaluate the effect of sonodynamic treatment on the main cancer hallmarks. The sonodynamic-treated group had a significant reduction (*p* ≤ 0.0001) in tumor size compared to the untreated group, and the Ala- and US-only treated groups, where a strong decrease (*p* ≤ 0.0001) in Ki67 protein expression was the most relevant feature of sonodynamic-treated cancer tissues. Moreover, oxidative stress was confirmed as the pivotal driver of the anticancer effect through cell cycle arrest, apoptosis, and autophagy; thus, sonodynamics should be explored further for cancer treatment.

## 1. Introduction

Sonodynamic therapy (SDT) is an anticancer and antibacterial approach [1,2], which relies on the synergism between ultrasound (US) and chemical compounds, known as “sonosensitizers”. Usually, SDT consists of the selective uptake of the sonosensitizer by cancer cells or a bacterial environment, and the subsequent US exposure, which leads to the generation of highly reactive cytotoxic agents, namely, hydrogen atoms, hydroxyl radicals, peroxyl and alkoxyl radicals and singlet oxygen, which damages cancer cells and bacterial cells [3,4,5].

Protoporphyrin IX (PpIX) is one of the most used photosensitizers in photodynamic therapy (PDT) for solid tumors; administering the heme precursor, 5-aminolevulinic acid (Ala), causes PpIX to selectively accumulate in cancer cells [6,7,8,9,10]. Although PDT with PpIX has been shown to be effective in various tumor models, the low penetration of light through tissues limits the synergistic effects between PpIX and light to treating only skin or superficial endoscopically accessible tumors [11].

Dissimilar to light, US can deeply penetrate biological tissues, due to its low coefficient of tissue attenuation, and, therefore, overcomes the main drawback of PDT [12]. Indeed, US energy absorption can cause tissue heating, which has been used in high intensity focused ultrasound (HIFU) therapy [13]. However, various studies have shown that therapeutic goals can also be achieved by taking advantage of non-thermal US effects, which can interact with cell membranes and activate specific chemical agents, such as PpIX, mainly through two different hypothesized mechanisms [14,15,16].

The first mechanism involves inertial acoustic cavitation that can occur if the amplitude of acoustic pressure is higher than the cavitation threshold in the tissue upon US exposure [17]. Inertial cavitation, thus, generates gas- or vapor-filled cavities into the milieu under US exposure, which initially increase in volume and then violently implode, creating so-called ‘hot spots’ where very high pressure and temperatures are restricted to an extremely small space. This unique condition is attained without affecting bulk pressure or temperature and, consequently, eliciting effects such as sonochemical reactions and sonoluminescence [18]. Inertial cavitation may then cause a transfer of energy able to cause electronic excitation in responsive chemical agents, namely, sonosensitizers [5].

The second hypothesis proposes that the intracellular sonosensitizer activation could be elicited by an US-mediated transfer of energy as intramembrane cavitation through the cell membrane in accordance with the bilayer sonophore (BLS) theory [16]. In other words, Krasovitski et al. proposed that the cell membrane, under suitable conditions, can transform the acoustic pressure of US into an intramembrane cavitation, generating, similar to inertial acoustic cavitation, intracellular submicron-sized gas bubbles which, upon collapsing, release extremely high energy and, possibly, sonoluminescence and cause an energy transfer, which could trigger electronic excitation in sonosensitive molecules [16,19].

Although the mechanism underlying SDT is still under debate, various in vitro and in vivo studies have been conducted with encouraging results, but there is still a wide gap between the bench and bedside [1,4,14,15,20,21]. This is also confirmed by the absence of clinical trials, and the only studies involving SDT, where cancer patients were enrolled, are case reports [1]. There are two case reports that involve breast cancer patients with positive outcomes, but SDT was always administered in combination with other anticancer treatments [22,23]. Therefore, to increase the number of case reports and to promote clinical trials (particularly in breast cancer), more preclinical experiments should be performed to prove the reliability, robustness, and effectiveness of SDT. To date, several in vivo SDT studies have been carried out against breast cancer, all with encouraging results, but some were in combination with PDT, and many with the aim of developing new sonosensitizers, and, thus, less suitable for a clinical trials evaluation [24,25]. This study, therefore, tries to show, for the first time, the possible efficacy and molecular features of SDT in a syngeneic breast cancer model with Ala as a sonosensitizer, a well know photosensitizer widely used in clinical application [26,27].

## 2. Results

### 2.1. SDT Effect on Tumor Growth

**Figure 1:**
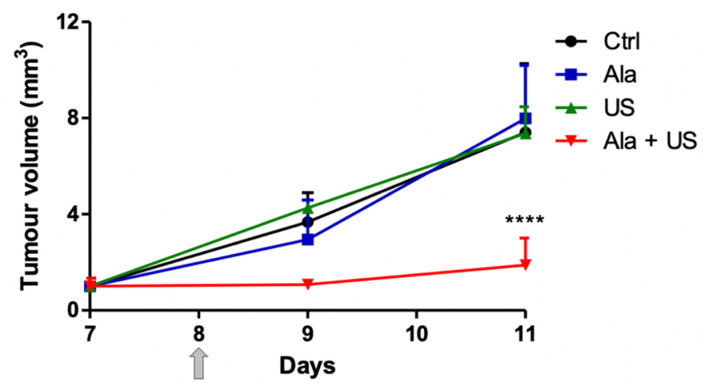
SDT effect on tumor growth. Control and experimental groups on day 8 (grey arrow) were either treated with a saline iv injection into the tail vein (0.5 mL), an Ala iv injection into the tail vein (375 mg/kg bw), US alone (1.5 W cm^2^, 1.8 MHz, 300 s), or Ala and US (1.5 W cm^2^, 1.8 MHz for 300 s, at 4 h after the Ala 375 mg/kg bw iv injection, SDT group). Tumor sizes were measured at 24 h (day 9) and 72 h (day 11) after treatment. Statistical significance vs. untreated rats (control, Ctrl): **** *p* ≤ 0.0001.

There were clearly no significant differences in tumor size between groups before treatment (day 7), while a decrease in tumor volume of about 70% at 24 h (day 9), and about 74.5% at 72 h (day 11) after treatment (Figure 1), was observed in the SDT-treated group. There was a statistically significant difference when the SDT animal group (1.89 ± 1.10 mm^3^) was compared to the control animal group (7.41 ± 2.85 mm^3^), the Ala (7.98 ± 2.22 mm^3^), and US (7.37 ± 1.11 mm^3^) animal groups at 72 h after treatment (day 11, *p* ≤ 0.0001, Figure 1).

### 2.2. SDT Effect on PARP Cleavage and CASP3 mRNA Expression

**Figure 2:**
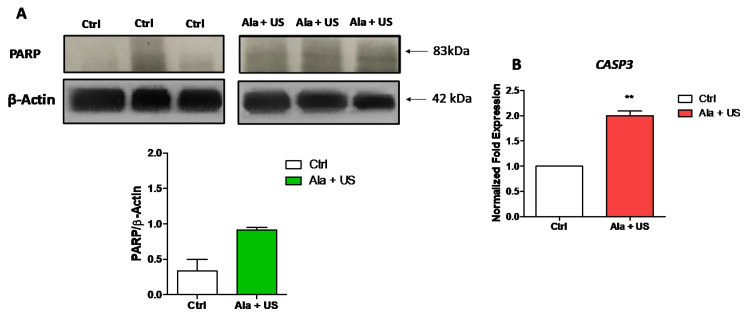
PARP cleavage and *CASP3* mRNA expression after SDT. Control and experimental groups were either treated with a saline iv injection into the tail vein (0.5 mL), an Ala iv injection into the tail vein (375 mg/kg bw), US alone (1.5 W cm2, 1.8 MHz, 300 s), or Ala and US (1.5 Wcm^2^, 1.8 MHz for 300 s, at 4 h after the Ala 375 mg/kg bw iv injection, SDT group). (**A**) Representative Western blots of PARP at 72 h after SDT indicate PARP cleavage to 83 KDa. Histograms show densitometric analysis normalized to the corresponding β-actin content. (**B**) *CASP3* mRNA expression by RT-PCR was analyzed at 72 h after SDT. *RRN18S* was used as a reference gene to normalize the data (mRNA levels were compared with those of the control group, stated as 1). Statistical significance vs. untreated tissue (Ctrl): ** *p* ≤ 0.01.

In order to verify whether Mat B III tumors in 12-week-old female Fisher 344 rats exposed to Ala (375 mg/kg bw, iv) and/or US (1.5 W cm^2^, 1.8 MHz for 300 s) expressed any hallmarks of apoptosis, i.e., specific cleavage of PARP into the 83 kDa fragment (due to caspase activation), Western blotting was carried out. PARP cleavage was evident at 72 h post-treatment (day 11) (while not statistically significant) in tumor tissue after SDT compared to the control animal group (Figure 2A).

To confirm the specific cleavage of PARP, and to better understand the apoptotic pathways triggered by different treatments, mRNA expression of the caspase CASP3 was investigated. Figure 2B shows that, at 72 h post-treatment (day 11), mRNA expression of CASP3 was upregulated by two-fold in tumor tissue after SDT treatment compared to tumor tissue in the untreated animal group (*p* ≤ 0.01).

### 2.3. SDT Effect on TP53 mRNA Expression and on Ki67 Protein Expression

**Figure 3:**
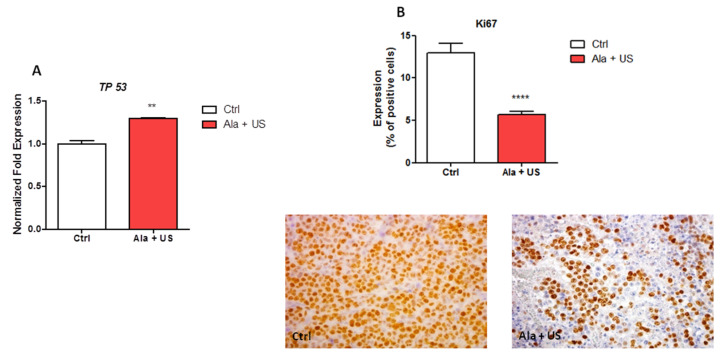
*TP53* mRNA expression and Ki67 protein expression after SDT. Control and experimental groups were either treated with a saline iv injection into the tail vein (0.5 mL), an Ala iv injection into the tail vein (375 mg/kg bw), US alone (1.5 W cm^2^, 1.8 MHz, 300 s), or Ala and US (1.5 W cm^2^, 1.8 MHz for 300 s, at 4 h after the Ala 375 mg/kg bw iv injection, SDT group). (**A**) Analysis of *TP53* mRNA expression by real-time RT-PCR at 72 h after SDT. *RRN18S* was used as a reference gene to normalize the data (mRNA levels were compared with those of the control group, which are stated as 1). (**B**) Ki67 protein expression by immunohistochemistry at 72 h after SDT (day 11), and representative Ki67 immunostaining (hematoxylin counterstain, original magnification ×400). Statistical significance vs. untreated tissue (Ctrl): ** *p* ≤ 0.01, **** *p* ≤ 0.0001.

Many studies have shown that the activity of the transcription factor p53 in cell cycle and apoptosis prevents tumor development, so we investigated p53 mRNA expression [28]. There was a statistically significant increase in *TP53* mRNA expression in tumor tissue at 72 h after SDT (day 11) compared to the control animal group (*p* ≤ 0.01, Figure 3A).

To confirm changes in the cell cycle arrest after SDT, Ki67 protein expression, a nuclear marker of cell proliferation, was studied by immunohistochemistry. A statistically significant decrease in Ki67 expression was observed in tumor tissue at 72 h after SDT treatment (day 11) compared to the control animal group (*p* ≤ 0.0001, Figure 3B).

### 2.4. SDT Effect on HIF-1α mRNA Expression and VEGF Protein Expression

To confirm the role of *TP53* gene activation in SDT-induced cell cycle blocking and apoptosis, the mRNA expression of *HIF-1α* and VEGF protein expression were investigated. The genetic activation of TP53 in cancer cells potently inhibits tumor angiogenesis (required for tumor growth) and inhibits *HIF-1α* mRNA and VEGF protein expression [29].

**Figure 4:**
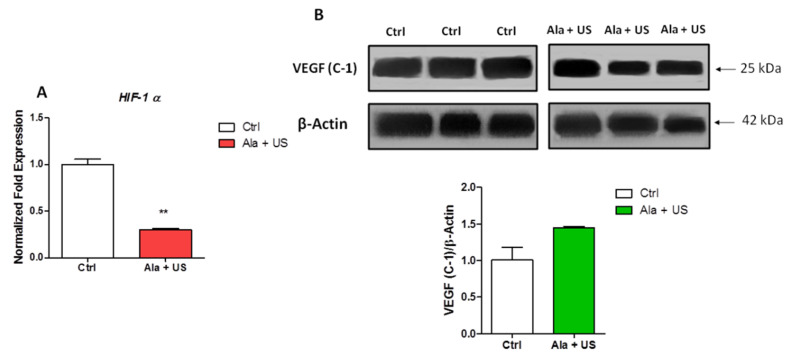
*HIF-1α* mRNA expression and VEGF protein expression after SDT. Control and experimental groups were treated with either a saline iv injection into the tail vein (0.5 mL), an Ala iv injection into the tail vein (375 mg/kg bw), US alone (1.5 W cm^2^, 1.8 MHz, 300 s), or Ala and US (1.5 W cm^2^, 1.8 MHz for 300 s, at 4 h after the Ala 375 mg/kg bw iv injection, SDT group). (**A**) Analysis of *HIF-1α* mRNA expression by real-time RT-PCR at 72 h after SDT. *RRN18S* was used as a reference gene to normalize the data (mRNA levels were compared with those of the control group, which are stated as 1). (**B**) Representative Western blots of VEGF protein expression 72 h after SDT. Histograms report densitometric analysis normalized for the corresponding β-actin content. Statistically significance vs. untreated tissue (Ctrl): ** *p* ≤ 0.01.

Our results showed a statistically significant downregulation of *HIF-1α* mRNA expression in tumor tissue at 72 h after SDT (day 11) compared to the control animal group (*p* ≤ 0.01, Figure 4A), and no statistically significant change in VEGF protein expression in tumor tissue at 72 h after SDT (day 11) compared to the control animal group (Figure 4B).

### 2.5. SDT Effect on NFE2L2 and NQO1 mRNA Expression

To kill cancer cells, SDT shifts the intracellular environment toward pro-oxidant conditions, due to reactive oxygen species (ROS) accumulation; we, thus, investigated the mRNA expression of oxidative stress-related genes, namely, *NFE2L2* and *NQO1* in tumor tissue at 72 h after SDT (day 11).

**Figure 5:**
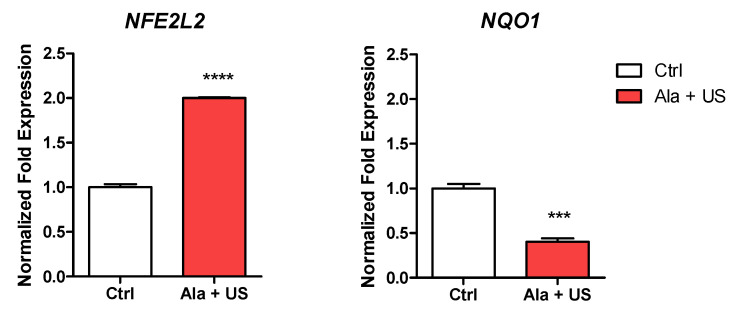
*NFE2L2* and *NQO1* mRNA expression after SDT. Control and experimental groups were either treated with a saline iv injection into the tail vein (0.5 mL), an Ala iv injection into the tail vein (375 mg/kg bw), US alone (1.5 W cm^2^, 1.8 MHz, 300 s), or Ala and US (1.5 W cm^2^, 1.8 MHz for 300 s, at 4 h after the Ala 375 mg/kg bw iv injection, SDT group). *NFE2L2* and *NQO1* mRNA expression was determined by real-time RT-PCR at 72 h after SDT. *RRN18S* was used as a reference gene to normalize the data (mRNA levels were compared with those of the control group, which are stated as 1). Statistically significance vs. untreated tissue (Ctrl): *** *p* ≤ 0.001, **** *p* ≤ 0.0001.

There was a statistically significant increase in *NFE2L2* mRNA expression in tumor tissue at 72 h after SDT (day 11) compared to the control animal group (*p* ≤ 0.0001, Figure 5). Conversely, there was a statistically significant decrease in *NQO1* mRNA expression in tumor tissue at 72 h after SDT (day 11) compared to the control animal group (*p* ≤ 0.001, Figure 5).

### 2.6. SDT Effect on LC3 A/B Protein Expression

As our previous results seemed to confirm that apoptosis and cell cycle arrest, through ROS production, play a role in cancer cell death caused by SDT, we also investigated protein expression of other cell death effectors such as autophagy, a catabolic process sometimes considered as a separate modality of programmed cell death, and a modulator of the anticancer immune response [30,31]. Therefore, LC3 A/B protein expression was investigated by immunoblotting, a reliable method for checking autophagy and autophagic cell death [32].

**Figure 6:**
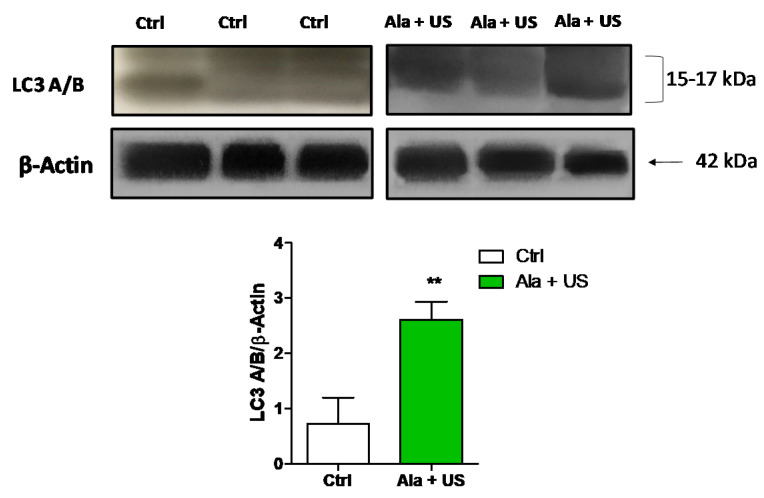
LC3 A/B protein expression after SDT. Control and experimental groups were either treated with a saline iv injection into the tail vein (0.5 mL), an Ala iv injection into the tail vein (375 mg/kg bw), US alone (1.5 W cm^2^, 1.8 MHz, 300 s), or Ala and US (1.5 W cm^2^, 1.8 MHz for 300 s, at 4 h after the Ala 375 mg/kg bw iv injection, SDT group). Representative Western blots of LC3 A/B protein expression at 72 h after SDT. Histograms report densitometric analysis normalized for the corresponding β-actin content. Statistical significance vs. untreated tissue (Ctrl): ** *p* ≤ 0.01.

Our data showed an increased expression of LC3 A/B protein expression in tumor tissue at 72 h after SDT (day 11) compared to the control animal group (*p* ≤ 0.01, Figure 6), suggesting a role for autophagy in sonodynamic cancer cell killing.

## 3. Discussion

In the 1990s, Umemura et al. and Tachibana et al. introduced the concept of SDT as an innovative anticancer approach to activate sensitizer cytotoxicity by US, and to overcome PDT’s main limitation, i.e., the low ability of light to penetrate human tissues [33,34]. Currently, even though many in vitro SDT studies have presented encouraging results in several cancer cell lines [3,35], few in vivo SDT studies have demonstrated significant tumor regression [1]. Therefore, more studies need to be carried out in vivo before SDT can be used as an adjuvant or replacement approach for traditional cancer treatment. We, therefore, carried out, in a syngeneic model of breast cancer, SDT with Ala as a sonosensitizer. We found that tumor growth was significantly reduced in the SDT animal group at 72 h after treatment (Figure 1), confirming the effectiveness of this anticancer approach in our syngeneic model of breast cancer [36,37,38,39].

In order to investigate the tumor size reduction in the SDT animal group compared to the control, we studied if SDT was able to trigger apoptosis (programmed cell death), typically inhibited in cancer, since many anticancer therapeutic strategies are related to the ability of the treatment to induce apoptosis [40]. Firstly, we investigated caspase-induced PARP cleavage, as caspase activation is one of the most common signal cascade pathways involved in apoptosis and is responsible for the cleavage of several key proteins required for cellular functioning and survival [41]. Therefore, PARP cleavage by caspases, resulting in various fragments with a specific molecular weight, is a hallmark of apoptosis [42]. Here, we observed an enhanced PARP cleavage in tumor tissue at 72 h after SDT treatment compared to the control animal group (Figure 2). Since this difference was not statistically significant, we investigated *CASP3* mRNA expression in the same tumor tissues, as this cysteinyl-aspartate protease is primarily responsible for PARP cleavage during cell death [43]. We found a statistically significant increase in *CASP3* mRNA expression in tumor tissue at 72 h after SDT treatment compared to the control animal group, suggesting that SDT induces apoptosis in solid tumors in vivo. This was supported, in vitro, by Li et al. in human pancreatic cancer cells and in vivo, by Foglietta et al. in a syngeneic rat model of breast cancer [14,36].

To investigate the mechanisms underlying SDT, and a possible role played by cell cycle arrest in reducing tumor growth after SDT, we studied mRNA expression of *TP53*, which codes for a nuclear DNA-binding phosphoprotein, involved in G_1_ cell cycle arrest [44]. In tumor tissue, 72 h after SDT, a statistically significant increase in *TP53* mRNA expression compared to the control animal group was observed, suggesting a link between the decrease in tumor growth and cell cycle arrest in SDT (Figure 3A).

To confirm that cell cycle progression could be a target in SDT, Ki67 expression was investigated, as this protein is tightly correlated with cell proliferation, and in some neoplasms, such as breast cancer, there is an inverse correlation between Ki67 expression and response to anticancer treatments [44,45,46,47,48]. Ki67 immunohistochemistry showed a statistically significant decrease in the expression of this protein in tumor tissue at 72 h after SDT compared to the control animal group, supporting the notion that SDT significantly decreased cell proliferation in the syngeneic model of breast cancer (Figure 3B).

Since SDT significantly decreased the cell proliferation rate, we also investigated if SDT could switch the syngeneic model of breast cancer towards a non-angiogenic phenotype, in order to avoid neoplastic growth and tumor progression. Therefore, we investigated *HIF-1α* mRNA and VEGF protein expression as *HIF-1α* activates gene transcription and stimulates angiogenesis by upregulating VEGF. In tumor tissue at 72 h after SDT, a statistically significant decrease in *HIF-1α* mRNA expression was detected compared to the tumor tissue in the control animal group (Figure 4). Furthermore, there was no evidence of VEGF protein upregulation between the two groups (Figure 4). This further highlighted the importance of p53 in SDT, as it has been previously reported that upregulating p53 inhibits *HIF-1α* expression [29].

Although the exact mechanism underlying SDT is still unclear, there is a wide consensus that ROS play a pivotal role in the sonodynamic-induced anticancer effect; therefore, to establish if a reduction in tumor growth was mainly attributable to oxidative stress in the syngeneic model of breast cancer subjected to SDT, mRNA expression of genes associated with oxidative stress, such as *NFE2L2* and *NQO1* genes, were investigated [4,21]. Our data indicated that, in tumor tissue subjected to SDT, the *NFE2L2* gene was upregulated (Figure 5), suggesting an increase in the half-life of Nrf2, a transcription factor able to translocate into the nucleus under stress, binding the antioxidant response element (ARE) in order to activate the transcription of cytoprotective genes, such as *NQO1, GST-1,* and *x-CT*, establishing the pro-oxidant conditions in tumor tissue after SDT [42]. However, we observed the downregulation of *NQO1* in tumor tissue after SDT, which apparently did not match with the upregulation of *NFE2L2* (Figure 5). It has also been shown that SDT-induced oxidative stress leads to the upregulation of p53 which, in turn, results in cancer cell cycle arrest and the downregulation of the Nrf2-dependent activation of antioxidant genes, such as *NQO1, GST-1,* and *x-CT* [49].

Finally, we examined LC3 A/B protein expression in order to determine if autophagy occurred following SDT in vivo, since Giuntini et al. [4] and Su et al. demonstrated that, in vitro, SDT caused cancer cell autophagy, dependent on ROS production [50]. Therefore, the observed SDT-induced autophagy, in vivo, suggested a role for SDT in modulating cancer cell death towards immunogenic cell death, which induces an adaptive immune response activation against cancer in immunocompetent hosts, resulting in a long-lasting protective antitumor immunity, a sort of ‘holy grail’ of anticancer therapeutics [51]. Our results showed a statistically significant increase in LC3 A/B protein expression in tumor tissue at 72 h after SDT compared to the control group (Figure 6). This outcome supports the idea that SDT may confer an immunological memory, able to preserve against tumor recurrence after the elimination of the primary tumor, as reported recently by Zhang et al., Chen et al. and Yin et al. [32,52,53].

## 4. Materials and Methods

### 4.1. Animals

Due to their immunocompetent system, syngeneic cancer models are consistent for the in vivo evaluation of new therapeutic approaches, being also time- and cost-effective models for obtaining reliable and robust translational data [54]. Therefore, based on our previous experience [36,55], we decided to use the syngeneic Mat B III breast cancer model. Inbred 8-week-old female Fisher 344 rats (Charles River Laboratories, Sant’Angelo Lodigiano, Italy) were housed in a specific pathogen-free environment at a 12 h light/dark cycle; rats had access to water and rodent laboratory chow ad libitum, and their weights were monitored. The procedures for animal care and handling were approved by the local “Animal Use and Care Committee”, in agreement with the European Directive 2010/63/EU. Moreover, the suitable number of animals per group was designated observing the guidelines for the statistical analysis of experiments involving laboratory animals [56].

The syngeneic rat mammary adenocarcinoma cell line, Mat B III (American Type Culture Collection, Manassas, VA USA), was cultured in McCoy’s 5A modified medium with 10% fetal bovine serum, 2 mM l-glutamine, 100 mg/mL streptomycin, and 100 units/mL penicillin (Sigma-Aldrich, Milano, Italy), keeping the incubator humidified, at 5% CO_2_ and 37 °C. For tumor induction, cells were detached from the flask, counted and orthotopically injected (1 *×* 10^6^ cells in 0.5 mL physiological saline) into the abdominal mammary fat pad of the inbred 12-week-old female Fisher 344 rats under isoflurane anesthesia.

### 4.2. Sonodynamic Treatment

At least four animals were randomly assigned to each experimental group, and three separate experiments were carried out according to our previous reports [36,55]. SDT was performed within 7 days, when the subcutaneous tumors reached approximatively 500 mm^3^ in volume. Ala powder (Sigma-Aldrich, Milano, Italy) was dissolved in physiological saline immediately before each administration (375 mg/kg body weight, bw).

Control and experimental groups were treated on day 8 with either a saline iv injection into the tail vein (0.5 mL), an Ala iv injection into the tail vein (375 mg/kg bw), US alone (1.5 W cm^2^, 1.8 MHz, 300 s), or Ala (375 mg/kg bw) and US (SDT group), with 1.5 W cm^2^, 1.8 MHz for 300 s, 4 h after the Ala iv injection into the tail vain.

At days 7, 9, and 11, all tumor masses were measured by caliper, calculating the tumor volume (V) by the formula V = 4/3πr^3^, with r as the mean of the two orthogonal radii. At the end of the study (day 11), all animals were sacrificed, and samples of tumor tissue were preserved in 10% buffered formalin for histology and in Allprotect Tissue Reagent (QIAGEN, Milano, Italy) for analyzing mRNA and protein expression.

**Figure 7:**
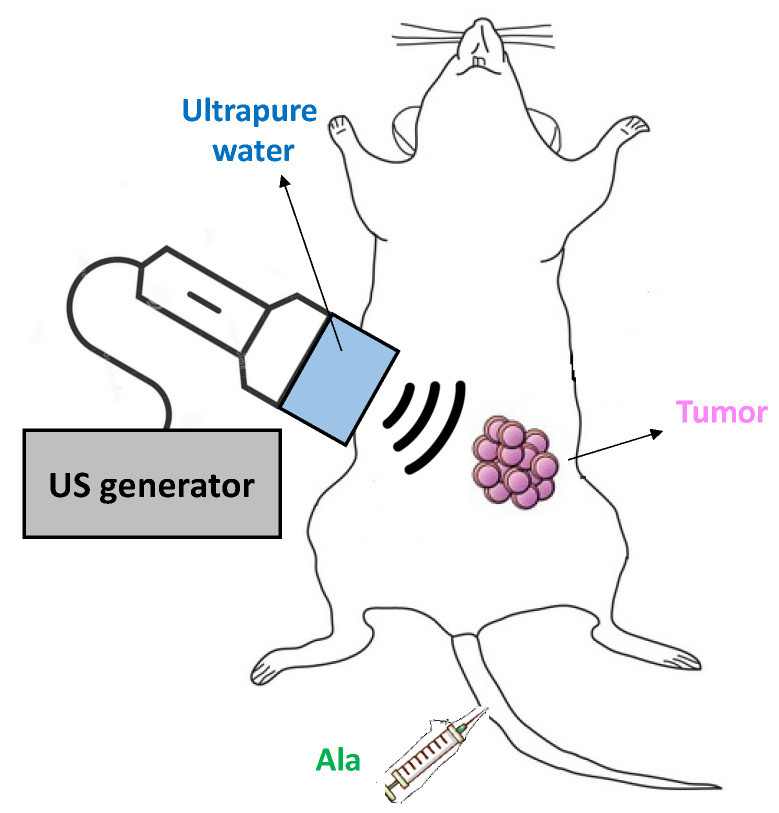
Representative scheme of SDT on rats. SDT-treated groups were subjected to Ala iv injection into the tail vein (375 mg/kg bw) and then exposed to US (1.5 W cm^2^, 1.8 MHz, 300 s), 4 h after the Ala iv injection.

SDT was performed by means of a plane wave transducer, working in continuous wave mode at 1.8 MHz frequency, which was connected to a function generator (Type 33250; Agilent, Santa Clara, CA, USA) and a power amplifier (Type AR 100A250A; Amplifier Research, Souderton, PA, USA). A proper mechanical adaptor was filled with ultrapure water, guaranteeing strong reproducibility of treatment conditions [57]; the distance from the transducer to the tumor was 2 cm, and US gel was applied between the adaptor and the naked rat skin. US treatment was performed at 1.5 W/cm^2^ for 300 s under subdued light, corresponding to a maximum root-mean-square acoustic pressure (rms) of 300 kPa. Before US exposure, rats were anesthetized with 1–2% isoflurane in air and O_2_, fixed in a supine position to a board with tumors facing upwards; US gel was applied to the shaved skin (Figure 7).

### 4.3. Western Blotting

Tumor samples were collected at 72 h post-treatment in Allprotect Tissue Reagent (QIAGEN) and preserved at −80 °C. Total proteins were extracted employing the AllPrep DNA/RNA/protein Kit (QIAGEN), and concentrations (μg/mL) were obtained using the Quant-iT RNA Assay Kit (Invitrogen, Milano, Italy) and the Qubit fluorometer (Invitrogen). Total proteins were denatured at 95 °C for 5 min by using a buffer (50 mmol/L Tris-HCl, pH 6.8, 100 mmol/L dithiothreitol, 0.10% bromophenol blue, 10% glycerol, 2% SDS), and then a final concentration of 30 μg of total protein was loaded onto an SDS-PAGE gel (Any kD™ Mini-PROTEAN^®^ TGX™ Gel, Bio-Rad, Segrate, Italy); proteins were transferred to a nitrocellulose membrane using the Trans-Blot^®^ Turbo™ Transfer System (Bio-Rad). Correct protein transfer was confirmed by incubating nitrocellulose membranes with Ponceau Red solution (Sigma-Aldrich). Membranes were then incubated at room temperature for 2 h with a Tris-buffered saline containing 0.05% TWEEM (Sigma-Aldrich) with 5% non-fat dry milk, and incubated overnight with the following primary antibodies: β-actin (Abcam, cat n° 8226), LC3A/B (Abcam, cat n° 128025), poly(ADP-ribose)polymerase (PARP, Abcam, cat n° 32138), and VEGF C-1 (Santa Cruz Biotechnology, Heidelberg, Germany, n° sc-7269). Following primary antibody incubation, peroxidase-conjugated IgG Abcam (Cambridge, UK) was used as the secondary antibody (goat anti-mouse, Abcam, cat n° 6789; goat anti-rabbit, Abcam, cat n° 97080, Cambridge, UK), and membranes were incubated for 1 h at room temperature. The Western blot was then detected using the chemiluminescent system (ECL, GE Healthcare, Milano, Italy), and band quantification was carried out by densitometric analysis using TotalLab Software, version 2006 (Nonlinear Dynamics, Newcastle, UK); data derived from the densiometric analysis of bands were normalized to the corresponding β-actin content.

### 4.4. RNA Isolation and SYBR Green Real-Time RT-PCR

Tumor samples were collected in Allprotect Tissue Reagent (QIAGEN) at 72 h after treatment, and preserved at −80 °C. The AllPrep DNA/RNA/protein Kit was used to obtain total RNA, and concentrations (μg/mL) were obtained using the Quant-iT RNA Assay Kit on the Qubit fluorometer. Moreover, total RNA 6000 Nano Kit on the Agilent 2100 Bioanalyzer (Agilent Technologies, Santa Clara, CA, USA) was used to analyze RNA sample integrity. Total RNA (150 ng) was reverse transcribed in a 20 μL cDNA reaction volume by the QuantiTect Reverse Transcription Kit (Qiagen, Milano, Italy). Real-time RT-PCR analysis was then performed using SsoFast EvaGreen on the MiniOpticon Real-Time RT-PCR system (Bio-Rad). A QuantiTect Primer Assay was used as the gene-specific primer pair for NQO1 (QIAGEN, QT00050281) coding for NAD(P)H quinone dehydrogenase 1; NFE2L2 (QIAGEN, QT00027384) coding for nuclear factor-erythroid 2-like 2; TP53 (QIAGEN, QT00060235) coding for tumor protein p53; HIF1A (QIAGEN, QT00083664) coding for hypoxia inducible factor 1 subunit alpha; APAF1 (QIAGEN, QT00092358) coding for apoptotic peptidase activating factor 1; CASP3 (QIAGEN, QT00023947) coding for apoptosis-related cysteine peptidase 3 (caspase 3); RRN18S (QIAGEN, QT00199367) coding for small subunit ribosomal RNA 18S. The ribosomal 18S and 28S RNA (RRNA18S) was used as a reference to normalize mRNA data. The PCR protocol has been previously reported [58], and data quantification analysis was performed using Bio-Rad CFX Manager Software version 1.6 (Bio-Rad). Real-time RT-PCR was performed by running each sample in duplicate for each group, according to three independent experiments. Values were then mediated and correlated to the housekeeping gene (RRN18S) and expressed as ratios compared to the untreated group (Ctrl), stated as 1.

### 4.5. Histopathological Analysis

Buffered formalin (10%) was used to fix tumor samples at 72 h post-treatment, which were, then, paraffin-embedded and cut to obtain 4 μm slide sections by using a microtome (Leica Microsystems, Wetzlar, Germany). Tumor slides were deparaffinized in xylene, rehydrated with alcohol and then stained with hematoxylin and eosin for histological examination by light microscopy (Leica DM600, Wetzlar, Germany). Moreover, selected slides were subjected to immunohistochemical analysis for Ki67, a nuclear protein that is a marker of cell proliferation, also used to categorize good and poor prognostic categories in invasive breast cancer [59]. A polyclonal antibody for Ki67 (dilution, 1:100; catalog n^o^ M7240; Dako, Santa Clara, CA, USA) was used according to the labeled streptavidin–biotin method (LSAB and System HRP Dako LSAB 2 System-HRP for use on rat specimens, Dako). Tumor sections were heated at 98 °C for 40 min in sodium citrate buffer (0.01 M, pH 6.0) for antigen retrieval. Endogenous peroxidase activity was quenched by incubating the specimen for 5 min in 3% hydrogen peroxide at room temperature. Finally, tumor slides were incubated overnight with the primary antibody in a humidified chamber at 4 °C, followed by sequential 10 min incubations with biotinylated-linked antibody and peroxidase-labeled streptavidin. Then, 3,3′-diaminobenzidine tetrahydrochloride (Sigma-Aldrich) was used to visualize the reaction, and Mayer’s hematoxylin was used as a counterstain. Quantification of IHC staining of Ki67 was carried out by randomly selecting six areas from each section, which were automatically quantified by Image-Pro Plus 6.0 software. Data are expressed as Ki67-positive cells in each sample.

### 4.6. Statistical Analysis

Results are expressed as the average values ± standard deviation (SD) of three separate experiments throughout and raw data are available in Supplementary Materials. Statistical analyses were performed using Prism 6.0 software (Graph-Pad, La Jolla, CA, USA). The Kruskal–Wallis test and the two-tailed Mann–Whitney U-test were used to calculate the threshold of significance. Statistical significance was set at *p* ≤ 0.05. Since statistical significance was not reported between the untreated (control) animal group vs. the US alone and Ala alone animal groups, the results only show comparisons between the SDT animal group vs. the control animal group, except for the SDT effect on tumor growth, where all groups are reported.

## 5. Conclusions

To conclude, this work suggested that SDT, which combines Ala with US treatment, is effective for tumor regression in a syngeneic model of breast cancer, leading to cell cycle arrest, apoptosis, and autophagy. Moreover, we showed the relevance of p53 upregulation upon SDT, facilitated by SDT-induced oxidative stress, and a possible role of SDT in the immune response against cancer. However, even though we showed that Ala, as a sonosensitizer, seemed to be effective in this preclinical study against breast cancer, there were still some technical limitations that must be addressed. In our opinion, the major concern about preclinical SDT studies is the complexity in experimental comparison among independent published reports due to the use of different sonosensitizers, custom-built US devices, and cavitation activity characterization. We, then, believe that the only way to overcome this issue is to increase in vivo studies with the same methods and goals. This could, therefore, encourage industries to develop specific US devices and clinicians to promote large-scale clinical trials to verify the safety and efficacy of SDT in patients.

## Data Availability

Data is contained within the article and Supplementary Material.

## Supplementary Materials

The following are available online at https://www.mdpi.com/article/10.3390/ph14100972/s1, Table S1 Raw data of PARP cleavage after SDT related to Figure 2; Table S2 Raw data of *CASP3* mRNA expression after SDT related to Figure 2; Table S3 Raw data of TP53 mRNA expression after SDT related to Figure 3; Table S4 Raw data of Ki67 protein expression after SDT related to Figure 3; Table S5 Raw data of *HIF-1α* mRNA expression after SDT related to Figure 4; Table S6 VEGF protein expression after SDT related to Figure 4; Table S7 Raw data of *NFE2L2* mRNA expression after SDT related to Figure 5; Table S8 Raw data of *NQO1* mRNA expression after SDT related to Figure 5; Table S9: Raw data of LC3 A/B protein expression after SDT related to Figure 6.
