# Supplementary material for: 5-Aminolevulinic Acid Triggered by Ultrasound Halts Tumor Proliferation in a Syngeneic Model of Breast Cancer
Source: Pharmaceuticals (Basel). 2021 Sep 25;14(10):972. doi: 10.3390/ph14100972 (PMC8540919; doi:10.3390/ph14100972)
Supplement: Supplementary file 1 [file pharmaceuticals-14-00972-s001.zip › pharmaceuticals-1356056-supplementary.pdf]

Supplementary Material

Table S1 Raw data of PARP cleavage after SDT related to Figure 2

**PARP/β-Actin**				
**Experiment 1**	**Animal 1**	**Animal 2**	**Animal 3**	**Animal 4**
**Ctrl**	0.500	0.267	0.279	0.600
**Ala + US**	1.230	0.870	1.350	0.760
**Experiment 2**	**Animal 1**	**Animal 2**	**Animal 3**	**Animal 4**
**Ctrl**	0.199	0.230	0.320	0.225
**Ala + US**	0.680	0.776	0.990	1.450
**Experiment 3**	**Animal 1**	**Animal 2**	**Animal 3**	**Animal 4**
**Ctrl**	0.567	0.360	0.278	0.230
**Ala + US**	1.200	0.450	0.678	0.543

Table S2 Raw data of *CASP3* mRNA expression after SDT related to Figure 2

CASP 3 (normalized fold expression)				
**Experiment 1**	**Animal 1**	**Animal 2**	**Animal 3**	**Animal 4**
**Ctrl**	0.980	1.011	0.990	1.099
**Ala + US**	1.870	2.760	1.760	1.850
**Experiment 2**	**Animal 1**	**Animal 2**	**Animal 3**	**Animal 4**
**Ctrl**	1.060	0.987	1.120	0.980
**Ala + US**	2.098	1.987	1.834	1.678
**Experiment 3**	**Animal 1**	**Animal 2**	**Animal 3**	**Animal 4**
**Ctrl**	1.087	1.090	0.987	0.870
**Ala + US**	1.090	2.987	1.911	2.700

Table S3 Raw data of *TP53* mRNA expression after SDT related to Figure 3

TP53 *(*normalized fold expression)				
**Experiment 1**	**Animal 1**	**Animal 2**	**Animal 3**	**Animal 4**
**Ctrl**	0.880	1.230	0.870	0.876
**Ala + US**	1.170	1.120	1.370	1.266
**Experiment 2**	**Animal 1**	**Animal 2**	**Animal 3**	**Animal 4**
**Ctrl**	0.870	1.100	0.988	0.865
**Ala + US**	1.230	0.987	1.450	1.780
**Experiment 3**	**Animal 1**	**Animal 2**	**Animal 3**	**Animal 4**
**Ctrl**	1.130	0.780	0.567	0.890
**Ala + US**	1.333	1.499	1.490	0.899

Table S4 Raw data of Ki67 protein expression after SDT related to Figure 3

**Ki67** (% of positive cells)				
**Experiment 1**	**Animal 1**	**Animal 2**	**Animal 3**	**Animal 4**
**Ctrl**	17.890	12.990	14.799	13.449
**Ala + US**	4.890	7.670	6.520	6.234
**Experiment 2**	**Animal 1**	**Animal 2**	**Animal 3**	**Animal 4**
**Ctrl**	13.950	11.570	9.590	17.990
**Ala + US**	6.780	5.150	4.600	5.780
**Experiment 3**	**Animal 1**	**Animal 2**	**Animal 3**	**Animal 4**
**Ctrl**	8.900	12.580	11.970	9.976
**Ala + US**	4.660	5.290	6.450	3.980

Table S5 Raw data of *HIF-1α* mRNA expression after SDT related to Figure 4

HIF1-α (normalized fold expression)				
**Experiment 1**	**Animal 1**	**Animal 2**	**Animal 3**	**Animal 4**
**Ctrl**	0.980	1.230	1.120	0.786
**Ala + US**	0.516	0.370	0.171	0.312
**Experiment 2**	**Animal 1**	**Animal 2**	**Animal 3**	**Animal 4**
**Ctrl**	0.999	1.112	1.340	0.777
**Ala + US**	0.333	0.307	0.416	0.208
**Experiment 3**	**Animal 1**	**Animal 2**	**Animal 3**	**Animal 4**
**Ctrl**	0.887	1.123	0.998	0.667
**Ala + US**	0.340	0.207	0.105	0.330

Table S6 VEGF protein expression after SDT related to Figure 4

**VEGF (C-1)/β-Actin**				
**Experiment 1**	**Animal 1**	**Animal 2**	**Animal 3**	**Animal 4**
**Ctrl**	0.700	0.900	0.800	0.770
**Ala + US**	1.400	1.480	1.350	1.602
**Experiment 2**	**Animal 1**	**Animal 2**	**Animal 3**	**Animal 4**
**Ctrl**	1.112	1.191	1.100	0.780
**Ala + US**	1.270	1.211	1.588	1.270
**Experiment 3**	**Animal 1**	**Animal 2**	**Animal 3**	**Animal 4**
**Ctrl**	1.300	1.400	0.991	1.119
**Ala + US**	1.357	1.649	1.664	1.501

Table S7 Raw data of *NFE2L2* mRNA expression after SDT related to Figure 5

NFE2L2 (normalized fold expression)				
**Experiment 1**	**Animal 1**	**Animal 2**	**Animal 3**	**Animal 4**
**Ctrl**	0.870	0.987	1.130	0.695
**Ala + US**	2.745	1.670	2.030	1.876
**Experiment 2**	**Animal 1**	**Animal 2**	**Animal 3**	**Animal 4**
**Ctrl**	1.220	0.983	0.699	1.149
**Ala + US**	1.983	1.335	1.786	2.650
**Experiment 3**	**Animal 1**	**Animal 2**	**Animal 3**	**Animal 4**
**Ctrl**	0.933	0.893	0.989	1.233
**Ala + US**	2.699	1.568	1.399	2.176

Table S8 Raw data of *NQO1* mRNA expression after SDT related to Figure 5

NQO1 (normalized fold expression)				
**Experiment 1**	**Animal 1**	**Animal 2**	**Animal 3**	**Animal 4**
**Ctrl**	1.150	0.860	0.954	1.100
**Ala + US**	0.580	0.450	0.339	0.277
**Experiment 2**	**Animal 1**	**Animal 2**	**Animal 3**	**Animal 4**
**Ctrl**	1.230	0.876	0.987	0.543
**Ala + US**	0.230	0.695	0.233	0.489
**Experiment 3**	**Animal 1**	**Animal 2**	**Animal 3**	**Animal 4**
**Ctrl**	1,098	1,357	0,997	0,854
**Ala + US**	0.490	0.279	0.330	0.399

Table S9 Raw data of LC3 A/B protein expression after SDT related to Figure 6

**LC3 A/B/β-Actin**				
**Experiment 1**	**Animal 1**	**Animal 2**	**Animal 3**	**Animal 4**
**Ctrl**	0.289	0.460	0.344	0.163
**Ala + US**	2.485	2.601	2.980	2.098
**Experiment 2**	**Animal 1**	**Animal 2**	**Animal 3**	**Animal 4**
**Ctrl**	0.648	0.563	0.448	0.555
**Ala + US**	2.178	2.250	2.147	2.493
**Experiment 3**	**Animal 1**	**Animal 2**	**Animal 3**	**Animal 4**
**Ctrl**	1.136	1.034	1.549	1.559
**Ala + US**	2.586	3.687	3.175	2.765
